# Anaesthetic Challenges in a Patient of Ludwig's Angina: A Case Report

**DOI:** 10.7759/cureus.30570

**Published:** 2022-10-21

**Authors:** Haneesha Movva, Karuna Taksande

**Affiliations:** 1 Department of Anaesthesiology, Jawaharlal Nehru Medical College, Datta Meghe Institute of Medical Sciences, Wardha, IND

**Keywords:** oedema and obstruction of the airway, second and third submandibular molars, trismus, dysphagia, ludwig's angina

## Abstract

Anaesthesiologists have difficult airways to manage while treating patients with severe neck infections. Ludwig's angina patients who are unable to adequately control their airways may pass away. In this article, we go through the anaesthetic management during an emergency drainage procedure for Ludwig's angina. In severe cases of Ludwig's angina, awake fiberoptic intubation under topical anaesthetic is the most effective strategy to maintain the airway. An awake tracheostomy may be the best alternative when awake fiberoptic bronchoscopy is not possible.

## Introduction

Ludwig's angina and deep neck infections are potentially fatal conditions. This is because of their propensity to result in oedema, distortion, and obstruction of the airway and the possibility that they could develop as a result of poor airway treatment. Patients can sometimes be treated in the early stages of the illness by being observed and receiving intravenous antibiotics. The airway must be protected and surgical drainage is necessary for advanced infections, though. A compromised airway is made worse by the discomfort, trismus, oedema, and displacement of the tongue. We give a quick overview of the various strategies for managing the airway before presenting a recent instance that was successfully handled at our hospital [1].

## Case presentation

**Figure 1:**
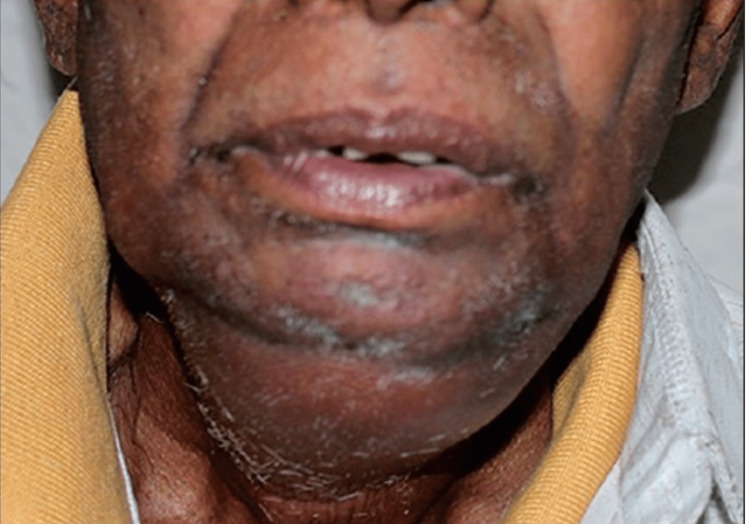
Preoperative frontal view

Upon arriving at the Department of Oral and Maxillofacial Surgery, a 65-year-old patient complained mostly of being unable to open the mouth, having pain and difficulty in swallowing, as well as swelling in their neck and lower jaw that had been present for the previous four days (Figure 1).

**Figure 2:**
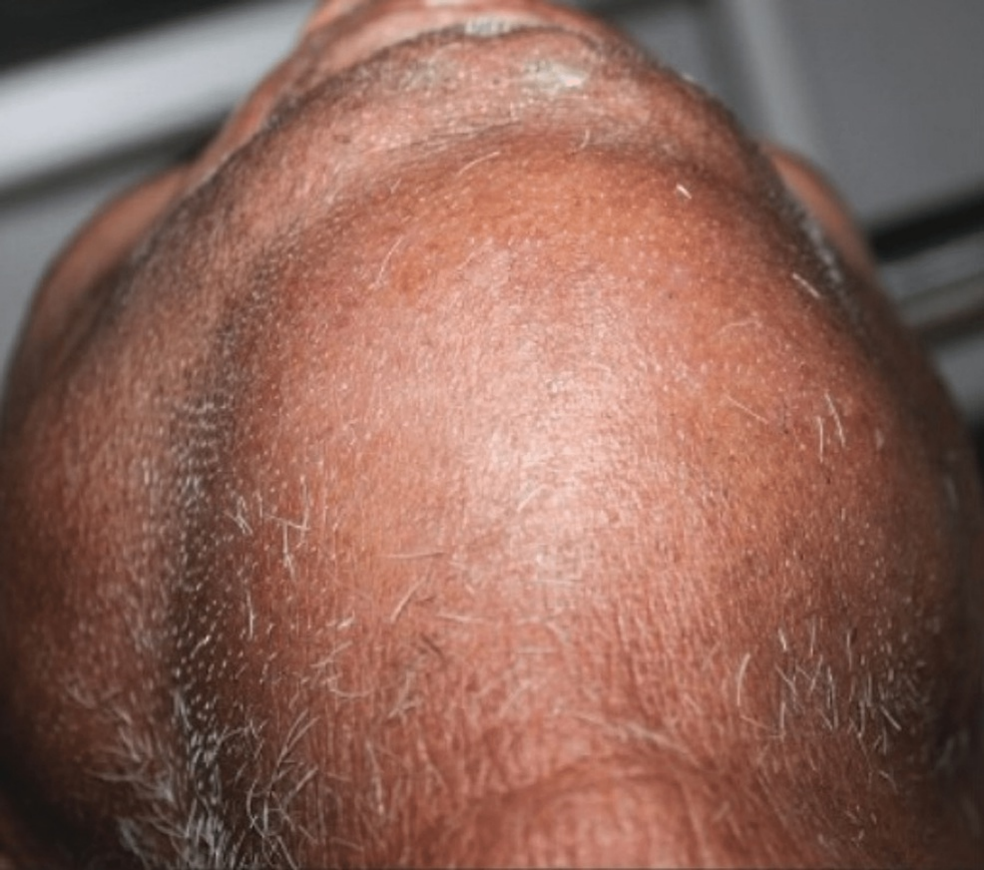
Preoperative neck view

Physically toxic, his vital signs were immediately monitored after a physical examination. A 100°F body temperature, 96 BPM pulse, a blood pressure of 120/70 mmHg, and 22 breaths per minute respiration were all present, and only allowed for a 1.5 cm mouth opening (interincisal distance). The submandibular and sublingual regions on both sides were involved in the extra-oral swelling, which was indurated and non-fluctuant (Figure 2).

**Figure 3:**
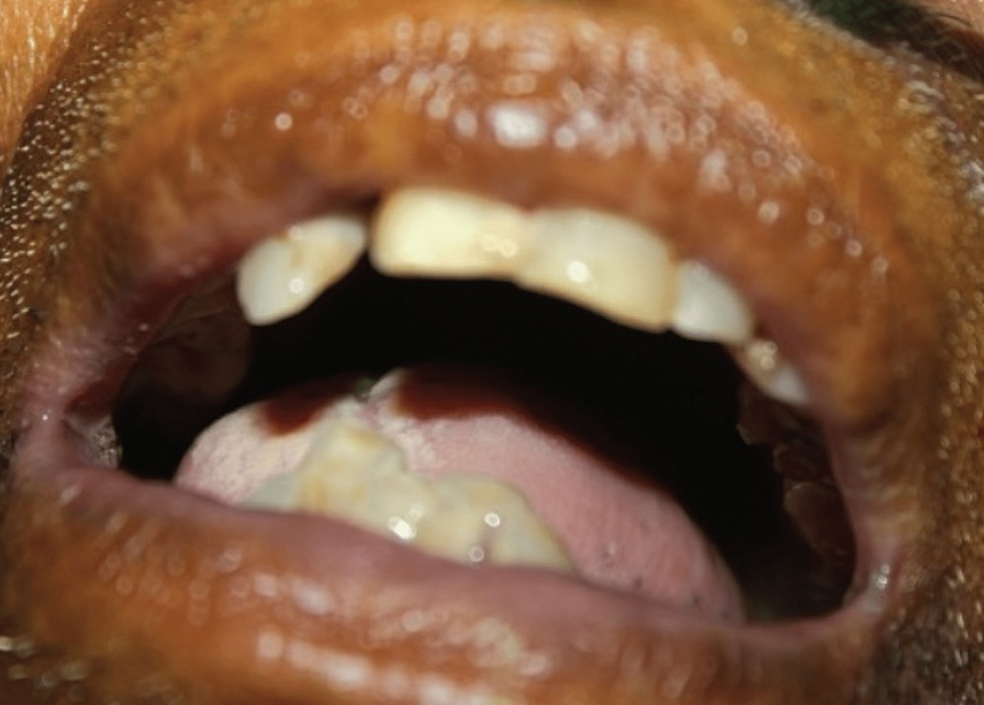
Preoperative mouth opening

Three days previously, an infected third molar had been removed. Ludwig's angina was diagnosed right away, and the patient was scheduled for surgical decompression under awake fiberoptic intubation and tracheostomy as a standby as they allowed for a 1.5 cm mouth opening (interincisal distance) (Figure 3).

The blood report was normal, with the exception of an increase in neutrophils, total white blood cells, and erythrocyte sedimentation rate (ESR). A difficult airway cart has been prepared since it was anticipated to be tough. After a successful bag and mask ventilation, direct laryngoscopy can also be conducted, but because fiberoptic bronchoscopy was a feasible alternative, we went with it. Fiberoptic intubation while awake was the plan, with tracheostomy as a backup. The patient gave informed consent for the tracheostomy and awake nasal intubation because they understood the procedure and the need for it.

**Figure 4:**
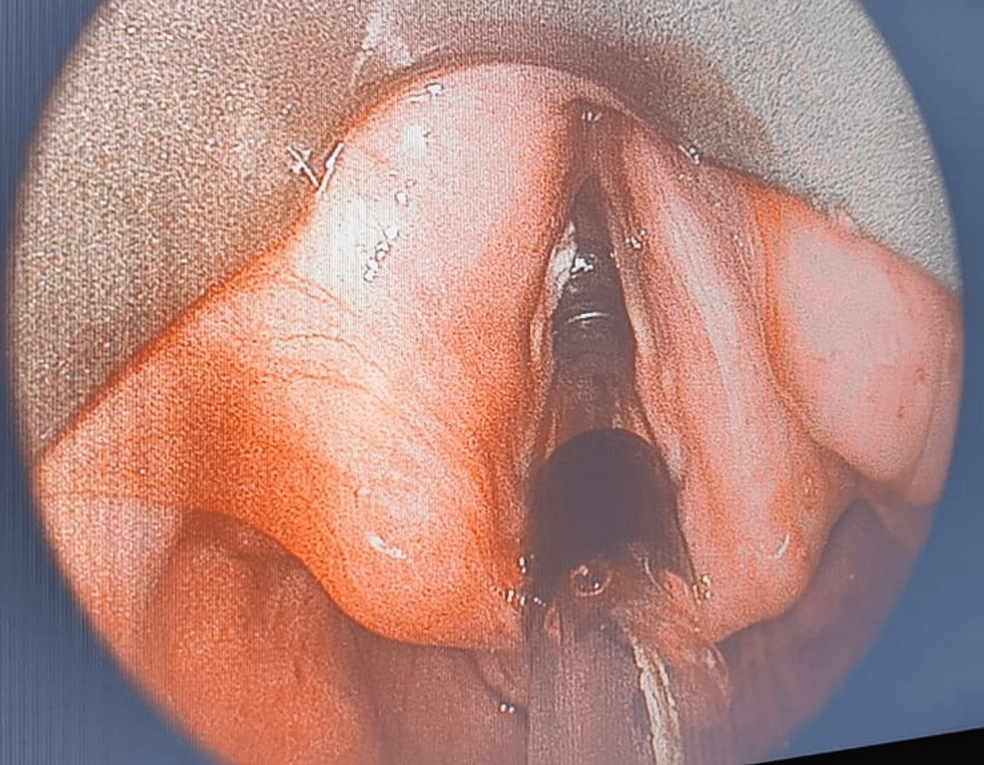
Fiber optic visualisation of the tip

As a premedication, the patient got 0.2 mg of injectable glycopyrrolate. Each nostril received one drop of 0.05% oxymetazoline nasal drops to help clear congestion, and each nostril received two drops of 4% lignocaine topical to numb the nasal mucosa. Lignocaine 2% and 10% were sprayed on the posterior pharyngeal wall to numb the base of the tongue and the pharyngeal walls. In the operation room, SpO2 (oxygen saturation), noninvasive blood pressure, and electrocardiography were all monitored. No oxygen was administered throughout the fiberscopy. After tracheal intubation was verified by fiberoptic visualisation of the tip inside the trachea (Figure 4) tube fogging, inability to vocalise, and end-tidal carbon dioxide, fentanyl 1-2 mcg/kg and propofol 1-2 mg were administered to induce anaesthesia. Vecuronium 0.1 mg/kg and dexamethasone 8 mg were also given. Anaesthesia was maintained with oxygen, nitrous oxide, and sevoflurane while using closed-circuit and intermittent positive-pressure breathing.

**Figure 5:**
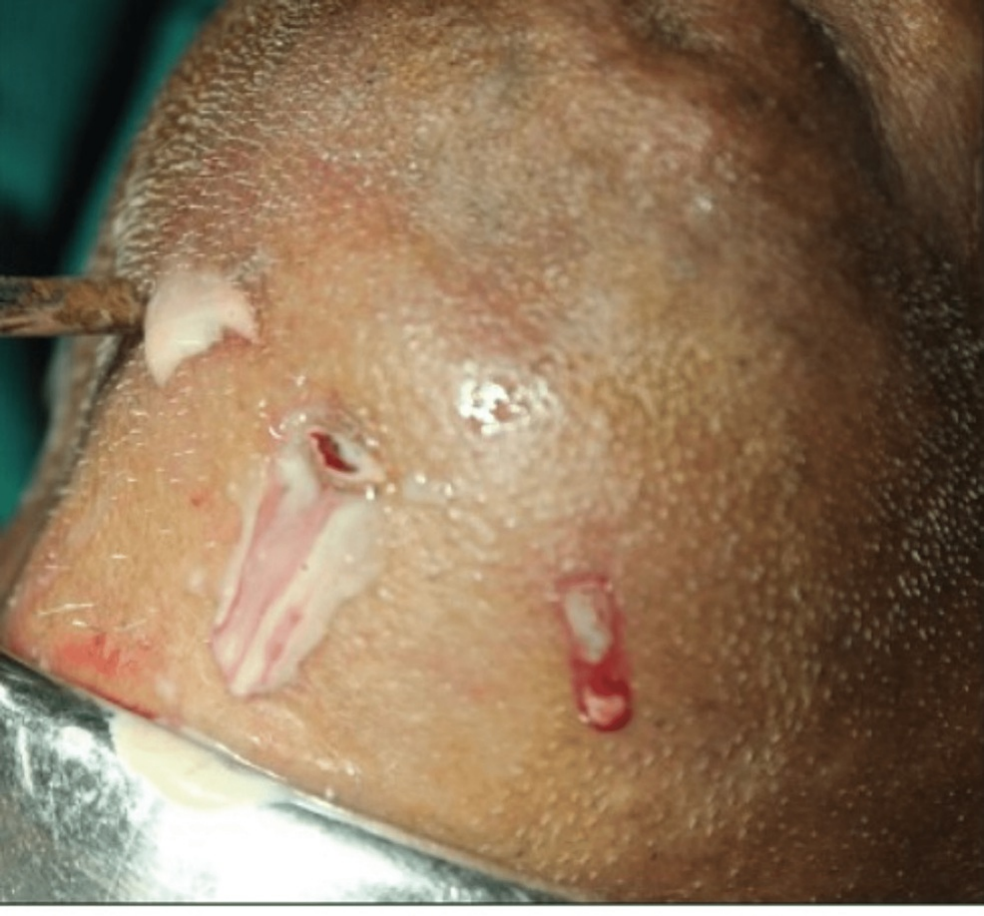
Draining pus

Throughout the treatment, the vital signs were steady. The submandibular space on both sides and the submental space were punctured separately. The pus was removed after the tissue gaps were opened up with sinus forceps (Figure 5). Normal saline was used to irrigate the incision, and a corrugated rubber drain was placed and fastened to the skin with sutures.

**Figure 6:**
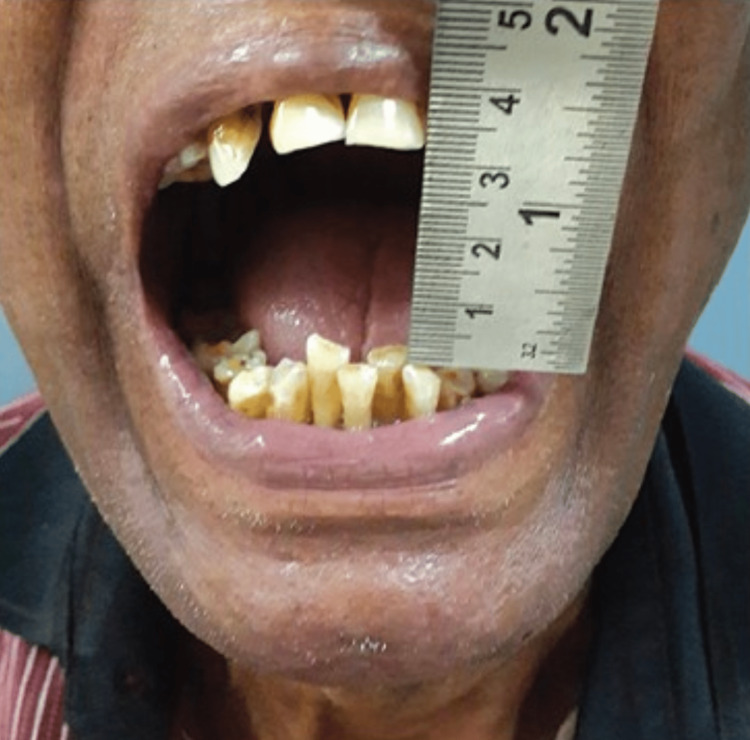
Postoperative mouth opening

The vital signs were stable during surgery. As a final reversal for any residual neuromuscular inhibition, 0.05 mg/kg of neostigmine was given. Due to periglottic oedema, the trachea was not extubated. Instead, the patient was moved to a post-anaesthesia care facility. While the endotracheal tube was still in place, the patient is placed on a T-piece, and 5 litres of oxygen were administered per minute. Sedation was produced by combining midazolam and fentanyl. The patient had a pulse rate of 75 beats per minute, a blood pressure measurement of 110/70 mmHg, and an oxygen saturation level of 97% the next morning. The neck oedema had diminished. A thorough oral suction was followed by extubation of the trachea. Although oedema had subsided and there was no cause to predict airway problems, the fibrescope was kept on standby. As a result, no elaborate tracheostomy or analogous procedure preparations were made. Following that the extubation went without an issue (Figure 6). The patient was discharged four days later [2].

## Discussion

Ludwig's angina is a relatively uncommon but rapidly progressing and frequently fatal gangrenous cellulitis and oedema of the soft tissues of the neck and floor of the mouth. Karl Friedrich Wilhelm von Ludwig provided the finest explanation of it in the year 1836. Ludwig's angina is hazardous because it can result in oedema and distortion, which obstruct the airway and make breathing difficult due to the tongue's elevation and posterior displacement. A slight infection sets off Ludwig's angina, which then causes induration of the upper neck, discomfort trismus, and tongue elevation. In the most typical odontogenic aetiology of Ludwig's angina, it typically develops in the second and third submandibular molars. The inferior submaxillary space and superior sublingual space are separated from the submandibular space by the mylohyoid muscle, allowing for unrestricted communication between the regions. Due to the potential for infection to spread through tissue spaces as a result, Ludwig's angina is bilateral. Additionally, the pharyngomaxillary and retropharyngeal areas are susceptible to infection [3]. As the infection travels further down into the mediastinum and pericardium, the involvement of the cardiovascular system may also be conceivable. Typical Ludwig's angina symptoms are present in our case.

Ludwig's angina can present with a variety of signs and symptoms, including tachycardia, fever, malaise, mild upper neck discomfort and swelling, pain in the lower posterior teeth, trismus, swelling in the mouth floor, superior/posterior displacement of the tongue, and trismus. Continuing swelling in the soft tissues of the neck also leads to increasing airway obstruction. Depending on how severe the condition is, dyspnea, dysphagia, and drooling may ensue. According to clinical observations, this obstruction is caused by the neck extending in the so-called "sniffing position". In certain circumstances, airway instrumentation has been postponed or avoided entirely by using intravenous dexamethasone and nebulized adrenaline to minimise upper airway oedema. Orotracheal intubation by direct laryngoscopy is challenging due to the distorted airway anatomy, tissue immobility, and constrained mouth access. In advanced cases, general anaesthetic induction is risky because it increases the risk of total airway closure, which prevents mask ventilation and intubation. The safest course of action is to secure the airway while still awake. Blind nasal intubation should be avoided since, in addition to having a high failure rate, it may result in aspiration, laryngospasm, airway oedema, pus rupture into the oral cavity, and catastrophic bleeding. Complete airway obstruction might result, which might call for an urgent cricothyrotomy. Tracheostomy was once regarded as the gold standard of care for creating a permanent airway. To prevent the risks of an emergency tracheostomy in a seriously compromised airway, it has been recommended that all patients with deep neck infections undergo elective awake tracheostomies. If the airway is not controlled right away, patients could develop tachypnea, stridor, cyanosis, and eventually pass away. Osteomyelitis and necrotizing fasciitis may potentially result from an infection of the nearby tissues. Airway obstruction was the main problem, as the patient's airway appeared to be compromised rather soon after presenting with tooth pain, even though no major involvement of the surrounding tissue was noticed during the course of treatment for our patient [4].

The criteria established by Ludwig and Grodinsky in 1939 serve as the foundation for the clinical diagnosis of Ludwig's angina. The illness was described as a submandibular cellulitis that could have spread to other neck spaces and caused swelling in the oral cavity's floor and superior/posterior displacement of the tongue. The infection spreads by continuity rather than through the lymphatic or circulatory system, and it rarely affects glandular tissues. Hematologic analysis and diagnostic imaging such as plain radiography, ultrasonography, CT and MRI may be additional valuable diagnostic testing (especially for severe cases which have the potential to involve multiple systems). Although crucial, these diagnostic procedures shouldn't postpone effective therapy [5]. Ludwig's angina is treated by immediately maintaining the patient's airway with endotracheal intubation or tracheostomy or cricothyroidotomy, and then by administering antibiotics and undergoing surgery. Since such severe infections cannot be treated with antibiotics alone. Surgical intervention, including an incision and drainage, is essential. Broad-spectrum antibiotics are used in antibiotic therapy to treat both aerobic and anaerobic bacteria, both of which may frequently be found in the oral cavity. In cases of Ludwig's angina, *Streptococcus *and *Staphylococcus *species are most frequently discovered. If medicinal care does not result in a considerable improvement or localised abscess formation is obvious, surgical intervention is necessary. This comprises decompression of the afflicted facial spaces through incision and drainage of purulence, if present [6].

Our patient was cared for by a multidisciplinary team made up of experts from otolaryngology, infectious disease, radiography, oral surgery, and dentistry. Endotracheal intubation, intravenous antibiotics, extraction of the mandibular second teeth, surgical incision, and drainage, both intraorally and extraorally, were all part of the treatment. From the submental and submandibular region, the drains stretched extraorally through the submental and submandibular spaces and into the oral cavity. Ludwig's angina must be treated with drains, however, if the drains are not positioned correctly, the bacterial infection may not completely resolve. For severe cases of Ludwig's angina, where substantial swelling has advanced to the lower neck region, a post-operative CT scan would be advantageous to ensure proper drainage of loculations and adequate placement of drains. It's also crucial to remember that infections are dynamic and need to be continuously evaluated until they are fully treated. Patients who develop strong infections frequently need to return to the operating room to have the incision and drainage repeated.

## Conclusions

In a somewhat typical age group, the current case illustrates a typical but severe case of Ludwig's angina. When many systems are affected, this is a life-threatening illness that necessitates early attention as well as an interdisciplinary team. Despite its rarity, Ludwig's angina is a dangerous condition that can be fatal. Poor dental hygiene may lead to the onset of Ludwig's Angina. The proper management of Ludwig's angina with significant soft tissue involvement necessitates early detection, airway care, broad-spectrum antibiotic medication, and surgical treatment.
